# Serum levels of stem cell factor for predicting embryo quality

**DOI:** 10.1038/s41598-024-61419-2

**Published:** 2024-05-22

**Authors:** Joanna Liss, Martyna Kuczyńska, Michał Kunicki, Krystian Zieliński, Damian Drzyzga

**Affiliations:** 1 Research and Development Center, INVICTA, Polna 64, 81-740 Sopot, Poland; 2 Department of Medical Biology and Genetics, University of Gdańsk, Gdańsk, Poland; 3 Department of Gynecological Endocrinology, Medical University of Warsaw, Warsaw, Poland

**Keywords:** Stem cell factor, SCF, Embryo quality, Endometriosis, Controlled ovarian hyperstimulation, Assisted reproductive treatment, Predictive markers, Endocrine system and metabolic diseases

## Abstract

We evaluated whether serum stem cell factor (s-SCF) levels just prior to ovulation induction could indicate the ability to develop a top-quality (TQ) blastocyst by day 5. We investigated patients with normal ovarian reserve (NOR), polycystic ovary syndrome (PCOS), diminished ovarian reserve (DOR), or mild endometriosis. Our pilot research suggests a correlation between s-SCF levels and the ability to form TQ blastocysts in patients with mild endometriosis. This significant statistical difference (*p* < 0.05) was noted between mild endometriosis patients for whom a TQ blastocyst was obtained and those for whom it was not possible, as measured on the 8th day of stimulation and the day of oocyte retrieval. The mean SCF levels in the serum of these women on the 8th day were at 28.07 (± 2.67) pg/ml for the TQ subgroup and 53.32 (± 16.02) pg/ml for the non-TQ subgroup (*p* < 0.05). On oocyte retrieval day it was 33.47 (± 3.93) pg/ml and 52.23 (± 9.72) pg/ml (*p* < 0.05), respectively.

## Introduction

Ovarian reserve and the mechanisms allowing for the continuous, gradual activation of primary follicles are determinants of the length of a woman’s reproductive lifespan. Measurement of serum anti-Müllerian hormone (AMH) levels and antral follicle count (AFC) are useful markers for predicting ovarian response to assisted reproductive treatment (ART)^1^. The success rate of a treatment depends not only on the quantity but also the quality of the retrieved oocytes^2–4^. However, biochemical markers that can predict the success of oocyte retrieval (with a high confidence level), expressed in terms of the quality of both retrieved oocytes and embryos, are still lacking. These markers can potentially limit empty retrieval or failed cycles. Thus, targeting folliculogenesis is one of the most promising approaches for the identification of efficient markers.

During folliculogenesis, primordial follicles develop through primary, preantral, antral, and preovulatory stages and eventually become capable of releasing an oocyte for fertilization^5,6^. The process of primordial follicle activation, which is the beginning of follicular development after puberty, determines ovarian reserve and reproductive lifespan^6^. Primary oocytes are arrested in the diplotene stage of meiosis until the primordial follicles begin to grow and finally reach the ovulatory stage^6–8^. It has been proven that a complex bidirectional signaling pathway between the oocyte and somatic cells, mediated by cytokines and growth factors, is required to activate and control the primordial follicle^9,10^.

The mammalian target of rapamycin (mTOR), a conserved serine/threonine kinase of the phosphatidylinositol kinase-related kinase family, is involved in follicle growth^11,12^. mTOR signaling in oocytes is important for the activation of primordial follicles, although it is not necessary for the transition from primordial to primary follicles^13^. In primordial follicle granulosa cells, mTOR signaling can either activate primordial follicles or impede their transition into primary follicles. Overexpression of one of the mTOR catalytic subunits (mTORC1) in primary follicles activates the oocyte through the cognate tyrosine kinase (c-Kit) receptor, thereby activating the PTEN/PI3K/Akt/FOXO3 signaling cascade^14^.

Stem cell factor (SCF) is a pleiotropic growth factor that influences target cells by binding to the c-Kit receptor. Both SCF and c-Kit are actively expressed across various developmentally distinct cell lineages during embryogenesis and adulthood^15^. SCF, secreted by granulosa cumulus cells (GCs), directly stimulates the growth and differentiation of oocytes and theca cells and increases steroid hormone production^16^. SCF, derived from GCs, binds to the c-Kit receptor on oocytes and stimulates the phosphoinositide 3-kinase (PI3K) signaling pathway^17^. Active PI3K catalyzes the conversion of phosphatidylinositol 4,5-bisphosphate (PIP2) to phosphatidylinositol 3,4,5-triphosphate (PIP3), thereby recruiting Akt kinase to the cell membrane, where it is activated through the phosphorylation of serine and threonine. The signal transmitted via this pathway is one of the key regulators of oocyte proliferation, differentiation, growth, and survival^12^. The cross-talk between GCs and oocytes involving the interaction of SCF and c-Kit receptor is relevant to the process of follicular development, from the recruitment of the primordial follicle to ovulation, and even the development of the early embryo^18^.

Salmassi et al. investigated the variability in serum concentrations of SCF (s-SCF) during the menstrual cycle in the process of follicular maturation, ovulation, implantation, and pregnancy, and in response to ovarian stimulation with recombinant follicle-stimulating hormone^15^. The authors demonstrated that SCF is produced during the follicular phase, immediately before the ovulatory phase, and may play an important role in folliculogenesis and ovulation. In addition, they demonstrated that serum s-SCF levels may be an indicator of successful ovarian stimulation, suggesting the possible role of SCF as a predictor of in vitro fertilization (IVF) outcomes^15^.

However, there is no consensus on how serum SCF levels could influence the ovarian response and therefore the outcome of controlled ovarian hyperstimulation (COH) in patients with different ovarian reserves^19–22^. More recently, the changes and correlations between AMH and SCF during COH have become a subject of study^23^. Furthermore, it is well known that AMH “suppresses” SCF levels, and the concentration of the latter positively correlates with oocyte maturation, embryo quality, and clinical pregnancy^22^.

Successful COH remains a challenge for clinicians because of various causes of infertility. It is unclear e.g. whether endometriosis primarily affects in vitro fertilization outcomes via oocyte quality. Patients with a low ovarian reserve had a significantly reduced number of follicles containing oocytes. Polycystic ovary syndrome (PCOS) contributes to the formation of immature follicles. In each of these cases, there may be abnormalities in the activation and maturation of the primary follicles in which SCF may be involved.

The limited scope of the study above indicates that there is still a need to identify a biomarker that would predict the qualitative success of a COH cycle, understood as the chance of proper embryo development before oocyte retrieval. Thus, in our study, we evaluated whether the SCF serum level just before the induction of ovulation could be a tool that would allow for the prediction of the qualitative success rate of COH.

## Results

The s-SCF level was measured in 195 serum samples collected during ovarian stimulation on days 1 and 8 and on the day of oocyte retrieval. SCF levels were juxtaposed with the formation of at least one top-quality (TQ) blastocyst and whether a clinical pregnancy was achieved. No significant differences in the Mann–Whitney U test (MWU test, *p* > 0.05) were observed in the mean s-SCF levels measured on the 1^st^ and 8^th^ day of stimulation and on the day of oocyte retrieval and the formation of non-top-quality (NTQ) and TQ blastocysts in all patients.

Next, the study group was divided into subgroups to assess the level of s-SCF depending on the cause of infertility. The subgroups distinguished were as follows: PCOS group (n = 24), diagnosed based on the Rotterdam criteria^24^, diminished ovarian reserve group (DOR) (n = 11), diagnosed according to biochemical and sonographic features collected during ovarian reserve testing, as suggested by the Bologna criteria^25^, mild endometriosis group (n = 10), confirmed by laparoscopy with histopathological verification^26^, and the normal ovarian reserve group (NOR) (n = 20) with “normal” body mass index (BMI) of 18.5–24.9 kg/m^2^, no more than two previous miscarriages and a serum basal anti-Müllerian hormone (AMH) level of 1.2–4.0 ng/ml.

The subgroups did not differ with respect to age (Table 1). The highest number of antral follicles was obtained in patients with PCOS (23.6 ± 11.40), while the lowest was in the endometriosis group (7 ± 4.5). The length of ovarian stimulation did not differ between the subgroups. In the PCOS group, a greater number of follicles on the oocyte retrieval day was observed (19.2 ± 10.1), while the number of mature MII oocytes and fertilized oocytes (9.3 ± 4.2 and 4.3 ± 3.0, respectively) was comparable to that in the normal ovarian reserve subgroup. The lowest number of TQ blastocysts on day 5 was observed in the endometriosis group (0.62 ± 1.1) and the highest in the NOR group (2.6 ± 1.9).

**Table 1:** Clinical outcomes after stimulation protocols in various infertility groups.

Parameter	NOR	DOR	PCOS	Endometriosis	*P*-value
Number of patients	20	11	24	10	–
Age (years)	33.2 (3.8)	34.7 (2.5)	32.5 (4.1)	33.9 (3.8)	0.42
AMH (ng/ml)	2.5 (0.7)^a^	0.9 (0.3)^ab^	6.8 (3.4)	2.1 (1.4)^ab^	0.00
AFC	13.6 (4.6)^a^	8.7 (3.4)^ab^	23.6 (11.4)	7 (4.5)^ab^	0.00
Length of ovarian stimulation (days)	8.4 (1.4)	9.7 (0.8)	8.9 (3.4)	9.8 (2.9)	0.37
Number of follicles on oocyte retrieval day	12.8 (7.2)^a^	6.5 (2.1)^ab^	19.2 (10.1)	7.1 (5.2)^ab^	0.00
Number of oocytes retrieved	13.2 (5.9)^ab^	6.2 (2.1)^c^	16.3 (7.9)^a^	7.6 (6.2)^bc^	0.00
Number of MII oocytes	9.2 (4.2)^a^	4.2 (1.9)^b^	9.3 (4.2)^a^	5 (4.3)^b^	0.00
Number of embryos (blastocysts on days 5–6)	4.5 (3.4)^a^	2 (1.3)^abc^	4.3 (3.0)^ab^	1 (1.4)^c^	0.01
Number of TQ blastocysts on day 5	2.6 (1.9)^a^	1.1 (1.2)^abc^	2.1 (1.6)^ab^	0.62 (1.1)^bc^	0.02

AFC, antral follicle count; AMH, anti-Müllerian hormone; DOR, diminished ovarian reserve; MII, metaphase II; NOR, normal ovarian reserve; PCOS, polycystic ovary syndrome; TQ, top-quality.

Data are presented as mean values with standard deviation (SD) in parentheses. The p-values determined by ANOVA are presented. Superscript letters (a, b, c) denote means that have no significant (p > 0.05) differences between groups in Tukey’s post-hoc test for multiple comparisons.

**Figure 1:**
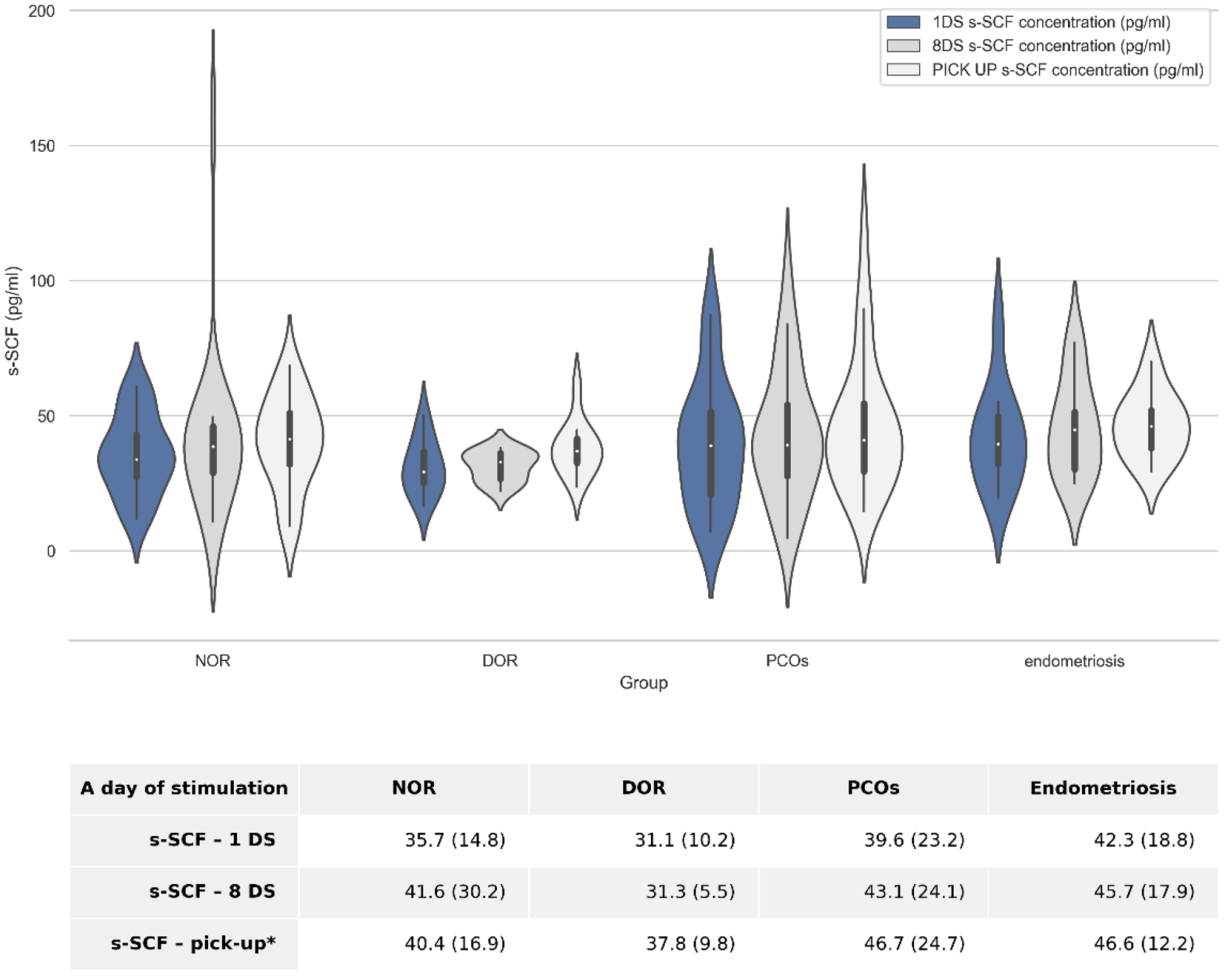
Comparison of s-SCF levels between study groups. Violin plots depict summary statistics and the density of each variable; they were not correlated with any of the clinical variables. The table below describes the mean s-SCF value (pg/ml) ± standard deviation (in parentheses) during controlled ovarian hyperstimulation. No significant differences in s-SCF levels were observed between the subgroups (Mann–Whitney U test, *p* > 0.05). DOR, diminished ovarian reserve; DS, day of stimulation; NOR, normal ovarian reserve; PCOS, polycystic ovary syndrome; s-SCF, serum stem cell factor; * day of oocyte retrieval (pick-up).

To verify whether the s-SCF level varied depending on the cause of the patient's infertility, we compared the s-SCF values determined on days 1 and 8 and on the oocyte retrieval day in the investigated subgroups (Fig. 1). No significant differences (MWU, *p* > 0.05) were observed between subgroups.

**Figure 2:**
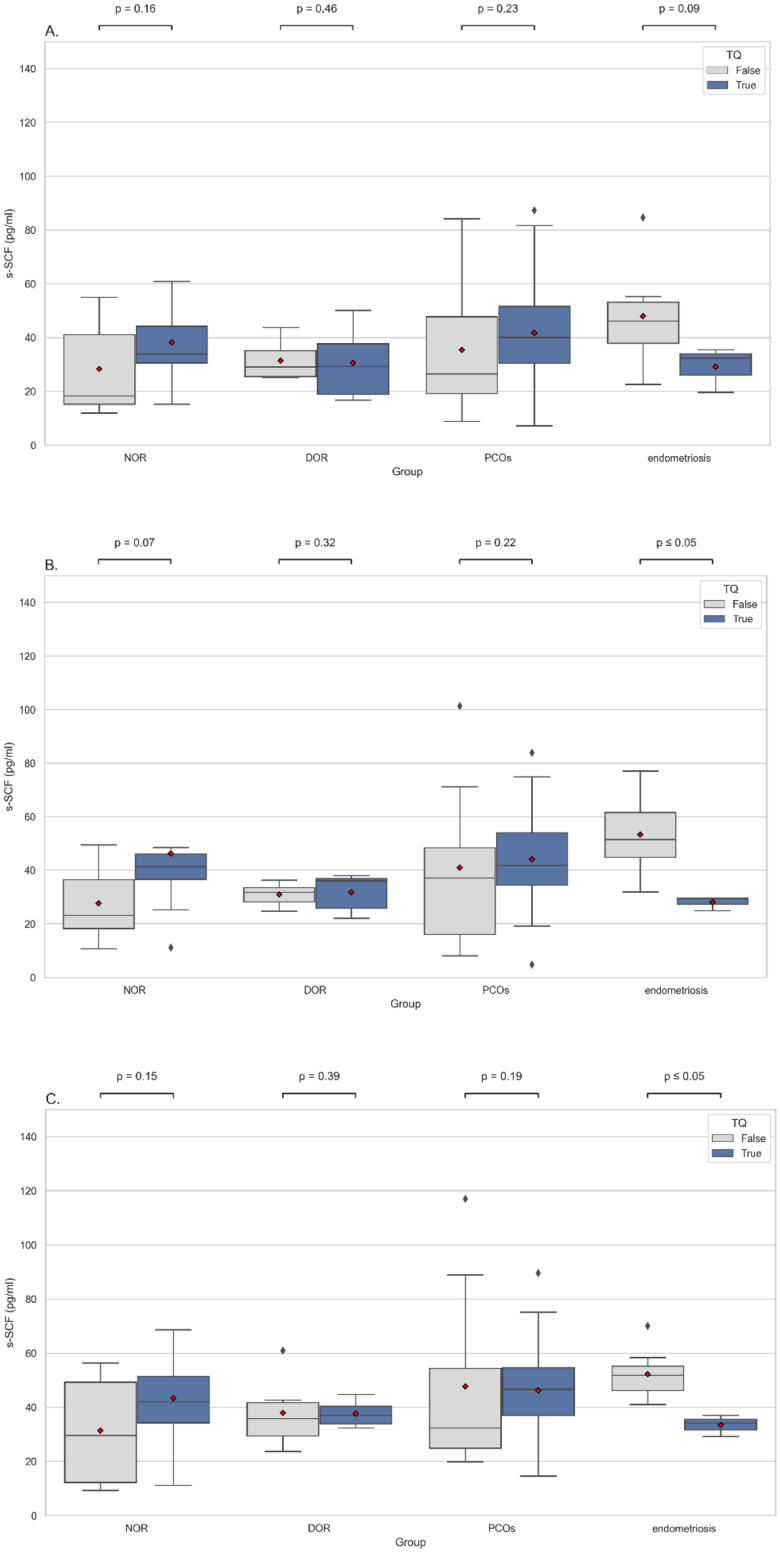
Comparison of s-SCF levels (pg/ml) between the study groups in relation to blastocyst quality. s-SCF levels were measured on the 1st day (**A**) and 8th day (**B**) of stimulation and on the oocyte retrieval day (**C**) in the studied groups. Patients within the groups were divided into subgroups depending on whether TQ blastocyt was obtained (true) or not (false). Box plots represent the distribution of variables. Means are marked with red points. Statistical significance was determined using the Mann–Whitney U test with *p*-value < 0.05 considered significant. DOR, diminished ovarian reserve; NOR, normal ovarian reserve; PCOS, polycystic ovary syndrome; s-SCF, serum stem cell factor; TQ, top-quality.

However, comparing the s-SCF levels on days 1 and 8 and the oocyte retrieval day with the chance to form top-quality blastocysts revealed an interesting relationship (Fig. 2). In the case of patients in the mild endometriosis group, the mean level of s-SCF was significantly lower on the 8th day of stimulation (28.1 pg/ml vs. 49.1 pg/ml; MWU test, *p* < 0.05) and on the oocyte retrieval day (33.4 pg/ml vs. 50.4 pg/ml; MWU test, *p* < 0.005) in samples from patients who had at least one TQ blastocyst on day 5 of culture (Fig. 2B and C).

There were no significant differences (MWU test, *p* > 0.05) in the s-SCF levels on day 1 (Fig. 2A) of stimulation between NTQ and TQ patients in PCOS, NOR, and DOR groups (41.1 pg/ml vs. 40.9 pg/ml; 34.8 pg/ml vs. 38.9 pg/ml and 32.3 pg/ml vs. 28.7 pg/ml respectively). The mean level of s-SCF in the endometriosis group patients was higher in the NTQ than the TQ subgroup and was 41.1 pg/ml and 29.1 pg/ml respectively. There were also no significant differences observed in the mean level of s-SCF in the NTQ and TQ subgroups on the 8th day of stimulation (Fig. 2B) and oocyte retrieval day (Fig. 2C) in PCOS, NOR, and DOR patients.

Our study suggests that a serum SCF level of less than 30 pg/ml during ovarian stimulation, especially on day 8th and on oocyte retrieval day in patients with mild endometriosis, may potentially suggest that there is a chance of obtaining at least one top-quality blastocyst and thus a chance of treatment success. More data, particularly considering the type of test used to assess SCF levels, are needed to confirm this relationship.

## Discussion

Many factors influence the successful development of embryos, with the quality of the mature ovarian oocytes playing a key role. Insufficiently developed cells can affect the success of fertilization and subsequent pregnancy. While assessing the levels of AMH, follicle-stimulating hormone (FSH), and antral follicle count through ultrasound examinations provides insight into the ovarian reserve, these measures do not directly inform us about the oocyte quality. The stem cell factor produced during the follicular phase may be a promising candidate for monitoring the activation, growth, and maturation of ovarian follicles. Its role in the regulation and growth of ovarian follicular function has been preliminary demonstrated^27–29^. The interaction of SCF with its receptor c-Kit appears to be an important pathway for the communication between oocytes and surrounding granulosa cells.

In our preliminary study, we explored s-SCF as a potentially useful marker for assessing the quality of formed embryos rather than as a putative indicator of the expected number of follicles and MII oocytes at the retrieval stage. This is one of the pioneering studies evaluating s-SCF levels as a potential early marker to predict the quality of retrieved oocytes and embryos. Here, we demonstrated a differentiated proportion of s-SCF levels during ovarian stimulation in the context of infertility. Previous studies have highlighted the role of SCF as an indicator of oocyte growth, particularly in patients with PCOS or diminished ovarian reserve^21^ or have focused on the level of SCF in follicular fluid^19,22^. Our study is the first to include and assess diverse groups of patients with various types of infertility.

Gizzo et al. showed a strong correlation between serum SCF levels and the number of mature oocytes in patients with diminished ovarian reserve and older than 43 years^19^. Their study concluded that, for these patients, an s-SCF assay could be useful during ovarian stimulation to assess the chances of success. An even more interesting finding of Gizzo et al. is that SCF concentration in both follicular fluid and serum remains stable regardless of the gonadotropin administration protocols used. This study indicated that serum SCF levels could be potentially applied to predict the number of embryos and pregnancy. It has already been proven that unceasing cell proliferation in the secretory phase of eutopic endometriosis is linked to deregulation of the c-Kit/SCF-associated signaling pathway^30^. This study showed that in patients with endometriosis, the expression of *c-kit* and Akt was elevated, suggesting that an increased number of available receptor binding sites could significantly lower the concentration of s-SCF^17^. This would trigger a cascade of events involving PI3K^12^. However, the assumptions presented here require further comprehensive research to elucidate the relationship between s-SCF levels, blastocyst quality, and clinical outcomes of embryo transfer.

For follicle development and maturation, a certain ratio between AMH and SCF levels is essential^21,31^. These two intraovarian factors play crucial roles in follicle recruitment and growth. However, details regarding the molecular mechanisms of these interactions are scarce. AMH downregulates SCF levels via the cyclic 3′,5′-adenosine monophosphate/protein kinase A (cAMP/PKA) signaling pathway^32^. Binding of AMH to its specific receptors on the surface of oocyte granulosa cells induces phosphorylation of cAMP response element-binding protein (CREB) and activates protein kinase A (PKA). Phosphorylated CREB interacts with the SCF promoter, significantly increasing its transcription^32^. Overexpression of AMH inhibits CREB phosphorylation and SCF expression. AMH also reduces the expression of aromatase, which is stimulated by FSH through the cAMP/PKA signaling pathway. The effect of these interactions is particularly evident in women diagnosed with PCOS who exhibit excessive AMH production but slower follicle growth^31^.

In our study, the PCOS subgroup showed a higher number of grown follicles than the other subgroups, although the numbers of MII cells and fertilized oocytes were comparable to those in patients with a normal ovarian reserve. Significant differences in SCF levels were found in patients with mild endometriosis who obtained top-quality embryos. The mean SCF level in the serum of these women was 30.23 (± 5.42) pg/ml. To date, no reports in the literature have confirmed our observations. Elevated SCF concentration is associated with endometriosis. Osuga et al. noted increased SCF levels in the peritoneal fluid of women at the early, mild stages (I/II) of endometriosis^33^. Conversely, women at more severe stages (III/IV) of endometriosis displayed SCF levels comparable to those in unaffected women, suggesting that heightened SCF levels may reflect the severity of endometriosis pathology. This may negatively influence IVF outcomes; therefore, further investigation of the relationship between SCF levels and IVF success is required.

It has been discussed that SCF is involved in the pathogenesis of endometriosis, particularly through the activation of mast cells. By binding to its c-Kit receptor, SCF could affect mast cells, leading to morphological changes and altered adhesion. Tryptase secreted by mast cells may presumably be associated with the activation of the protease-activated receptor 2 (PAR2) in endometrial cells^34^. PAR2, a member of the transmembrane G protein-coupled receptors, could act via MAPK, PI3K/Akt, or protein kinase C to promote the proliferation of endometriotic stromal cells^35^. Mast cell tryptase might accelerate endometriosis by increasing the number of endometriotic stromal cells and IL-6 and IL-8 secretion. The postulated molecular cascade is as follows: SCF stimulates mast cells to release tryptase; then, PAR2 is activated in endometriotic cells; these cells proliferate and secrete IL-6 and IL-8. IL-8 is a chemoattractant for neutrophils that migrate to endometriotic lesions^34^. Serine proteases released from neutrophils stimulate continues proliferation of endometrial cells, thereby contributing to disease progression. Chronic inflammation, particularly in the pelvic area, may indirectly affect the quality of oocytes. Patients with advanced endometriosis are characterized by a significantly reduced expression of aromatase^36^, redirecting our attention to cAMP/protein kinase A signaling, a probable point of interaction between AMH, FSH, E2, and SCF.

The strength of our study lies in its novelty: it is the first to assess SCF levels in patients with various types of infertility. Our findings highlight the variability in SCF levels according to infertility cause, particularly in patients with mild endometriosis. However, our findings are preliminary and further in-depth research is required. We are aware that our considerations are purely scientific but suggest a new direction for future studies. Until a direct link is established between SCF levels and both blastocyst quality and clinical outcome of an assisted reproductive procedure, it remains challenging to make key decisions (e.g., discontinuation of hormone stimulation) based on the SCF levels alone. Should our hypothesis be confirmed, serum SCF levels could inform clinical decisions regarding the collected oocytes such as wheather to fertilize or freeze them and wheather to repeat ovarian stimulation to collect oocytes with better prospects for resulting in pregnancy. It is also worth noting that our research contributes to a deeper understanding of the biochemical pathways involved in oocyte recruitment and maturation.

The design of the current study also had limitations. This study included a limited number of participants, with only 65 subjects undergoing in vitro procedures. Future studies should be conducted using larger cohorts. Patients with more advanced endometriosis should also be included, as our study focused on patients in the early stages of the condition. A detailed history of ovarian surgery or inflammatory diseases in the pelvic cavity should also be included to deeply analyze the impact of serum SCF on embryo formation. We also acknowledge that integration of preimplantation genetic testing for aneuploidy (PGT-A) could enrich our understanding of the embryo formation process. In our study, blastocyst classification was based on Gardner's metric. Although this metric is widely used in embryological laboratories as the primary tool for morphological assessment of developing blastocysts and is considered a universal approach for interpreting morphological data, using more advanced techniques to assess blastocyst development (e.g., application of a time-lapse system) could broaden the scope of evaluating data. Such an approach warrants consideration in further studies to explore the relationship between SCF levels and the chance of obtaining top-quality embryos.

## Conclusions

Our preliminary study suggests that serum SCF levels below 30 pg/ml during ovarian stimulation (day 8^th^ and on oocyte retrieval day) in patients with mild endometriosis could potentially be a predictor of the chance of obtaining at least one top-quality blastocyst on day 5 of growth, and therefore indicate the potential for the treatment success. More data are needed to confirm the link between s-SCF levels and formation of top-quality blastocysts in patients with endometriosis at different stages of the disease.

## Methods

### Patients

The study included 65 female patients with a mean age of 33.7 ± 4.2 years who underwent ovarian stimulation for fresh non-donor intracytoplasmic sperm injection (ICSI) treatment at Invicta Fertility Clinics from August 2019 to March 2020. All patients underwent pretreatment assessment of baseline ovarian reserve between days 2 and 5 of their cycle. The average serum FSH level was recorded at 7.3 ± 3.4 mIU/ml, and the AMH at 3.7 ± 3.2 ng/ml. The sonographic measurement of the antral follicle count (AFC) was determined to be on average 15.3 ± 9.9. Patients with abnormal karyotypes or inherited genetic diseases, severe male factor infertility, and cases in which oocyte retrieval was canceled due to an insufficient ovarian response were excluded from the study. Following our internal protocols, all patients upon admission provided written informed consent for the processing of their data in compliance with applicable privacy laws.

### Controlled ovarian hyperstimulation (COH)

Menopausal gonadotropins were used in monotherapy as described elsewhere^37^. All women were treated with a long agonist protocol starting with oral contraceptives (OCs) (Rigevidon, Richter Gedeon, Warsaw, Poland) from the 2nd to the 5th day of the cycle. Triptorelin acetate 0.1 mg (Gonapeptyl, Ferring, Saint-Prex, Switzerland) was administered 14 days after the initiation of the OCs. Fourteen days later (7 days after the end of OC administration), urinary gonadotropins (Menopur, Ferring, Saint-Prex, Switzerland) were administered for ovarian stimulation. Follicular growth was monitored on day 8 of COH using transvaginal ultrasound, and assays evaluating serum estradiol (E2), progesterone (P), and luteinizing hormone (LH) levels were performed until pituitary ovarian downregulation was reached (i.e., E2 concentration < 50 pg/ml). Follicular growth was stimulated by administering FSH (Menopur, Ferring, Saint-Prex, Switzerland), considering individual endocrine response as well as ovarian reaction estimated as the presence of at least an 18-mm-diameter follicle. Oocyte retrieval under the guidance of transvaginal ultrasound was performed 36–38 h after ovulation induction with human chorionic gonadotropin (hCG) injection.

### Serum sample collection

Serum samples from each patient were collected during routine preprocedural blood checks on the 1st and 8th day of stimulation, and on the day of oocyte retrieval. No additional interventions were performed to obtain samples. Following routine hormone analysis, serum samples were frozen and stored at − 80 °C until SCF level analysis.

### ELISA for SCF measurements

The concentration of s-SCF was determined using a commercially available enzyme-linked immunosorbent assay ELISA MAX™ Deluxe Set Human Free SCF kit (BioLegend, San Diego, USA, Cat. No. 442504). The expected minimum detectable concentration of free SCF in this set was 4 pg/ml. The cross-reactivity with mouse SCF was approximately 16% of that with human SCF at sample concentrations of 7.8–500 pg/ml. Recombinant human c-Kit did not cross-react in this assay. No cross-reactivity was observed when this kit was used to analyze the 74 other human/mouse recombinant chemokines and cytokines. All procedures were performed according to the manufacturer’s instructions.

### Oocyte and embryo assessment

Oocyte retrieval, ICSI procedures, and embryo morphology assessments were performed as previously described^38^. The top-quality (TQ) embryo category, that is, those exhibiting good morphological parameters, included day 2, day 3, and day 4 stage embryos with scoring class 1 or 2 (A or B, equivalent to the Gardner scale). TQ blastocysts were embryos that qualified as TQ and reached min stage 3 of development with additional trophectoderm (TE) and inner cell mass (ICM) scores of min 1 or 2. Embryos not meeting these criteria were described as poor, that is, non-top-quality (NTQ). The NTQ embryos included embryos with different than expected, at specific observation time points, number of blastomeres, and morphological features at a score of 3^39^.

### Statistical analysis

Statistical analysis was performed using the STATISTICA (v.13.1) software (StatSoft Power Solutions, Inc.) and Python (v. 3.8.5) package SciPy (v. 1.6.1). Normality was tested using the Shapiro–Wilk test. In general, for pairwise comparisons between two groups, the Mann–Whitney U (MWU) test was used. For analyses involving more than two groups, one-way ANOVA with Tukey’s post-hoc test for multiple comparisons was conducted. For all tests, a *p*-value < 0.05 was considered statistically significant. Figures were created using Seaborn (v.0.11.2) and Matplotlib (v.3.50) libraries in Python.

### Ethics declarations

The authors assert that all procedures contributing to this work complied with the ethical standards of the relevant national and institutional committees on human experimentation and the Helsinki Declaration of 1975, as revised in 2008. This study was approved by the Ethics Committee of the Okręgowa Izba Lekarska w Gdańsku—KB-41/21. Written informed consent was obtained from all participants enrolled in this study, and they agreed to use their biological samples for research.

## Data Availability

Data presented in this study are available upon request from the corresponding author.

## References

[1] Gizzo S (2014). Ovarian reserve test. Reprod. Sci..

[2] Azziz R (2016). Polycystic ovary syndrome. Nat. Rev. Dis. Primers.

[3] Rienzi L, Balaban B, Ebner T, Mandelbaum J (2012). The oocyte. Hum. Reprod..

[4] Vermey BG (2019). Is there an association between oocyte number and embryo quality? A systematic review and meta-analysis. Reprod. Biomed .Online.

[5] Albertini D, Combelles C, Benecchi E, Carabatsos M (2001). Cellular basis for paracrine regulation of ovarian follicle development. Reproduction.

[6] Hirshfield AN (1991). Development of Follicles in the Mammalian Ovary.

[7] Borum K (1961). Oogenesis in the mouse. Exp. Cell Res..

[8] Beaumont HM, Mandl AM (1962). A quantitative and cytological study of oogonia and oocytes in the foetal and neonatal rat. Proc. R. Soc. Lond. B Biol. Sci..

[9] Hutt KJ, McLaughlin EA, Holland MK (2006). KIT/KIT ligand in mammalian oogenesis and folliculogenesis: Roles in rabbit and murine ovarian follicle activation and oocyte Growth1. Biol. Reprod..

[10] Skinner MK (2005). Regulation of primordial follicle assembly and development. Hum. Reprod. Update.

[11] Guo Z, Yu Q (2019). Role of mTOR signaling in female reproduction. Front. Endocrinol. Lausanne.

[12] Makker A, Goel MM, Mahdi AA (2014). PI3K/PTEN/Akt and TSC/mTOR signaling pathways, ovarian dysfunction, and infertility: An update. J. Mol. Endocrinol..

[13] Adhikari D (2010). Tsc/mTORC1 signaling in oocytes governs the quiescence and activation of primordial follicles. Hum. Mol. Genet..

[14] Vanhaesebroeck B, Guillermet-Guibert J, Graupera M, Bilanges B (2010). The emerging mechanisms of isoform-specific PI3K signalling. Nat. Rev. Mol. Cell Biol..

[15] Salmassi A (2011). Circulating concentration of stem cell factor in serum of stimulated IVF patients. Reprod. Biomed. Online.

[16] Høyer PE, Byskov AG, Møllgård K (2005). Stem cell factor and c-Kit in human primordial germ cells and fetal ovaries. Mol. Cell Endocrinol..

[17] Reddy P (2005). Activation of Akt (PKB) and suppression of FKHRL1 in mouse and rat oocytes by stem cell factor during follicular activation and development. Dev. Biol..

[18] Figueira MI, Cardoso HJ, Correia S, Maia CJ, Socorro S (2014). Hormonal regulation of c-KIT receptor and its ligand: Implications for human infertility?. Prog. Histochem. Cytochem..

[19] Gizzo S (2016). Serum stem cell factor assay in elderly poor responder patients undergoing IVF: A new biomarker to customize follicle aspiration cycle by cycle. Reprod. Sci..

[20] Josso N (2019). Women in reproductive science: Anti-Müllerian hormone: A look back and ahead. Reproduction.

[21] Liu X-H, Wu X-H, Yang S (2019). Changes and correlations of anti-Müllerian hormone and stem-cell factors in different ovarian reserve patients during GnRH-antagonist protocol and the effects on controlled ovarian hyperstimulation outcomes. Arch. Gynecol. Obstet..

[22] Tan J (2017). Increased SCF in follicular fluid and granulosa cells positively correlates with oocyte maturation, fertilization, and embryo quality in humans. Reprod. Sci..

[23] Zhang J-F, Yu C-M, Yan L-L, Ma J-L (2018). Effect of anti-mullerian hormone on stem cell factor in serum, follicular fluid and ovarian granular cells of polycystic ovarian syndrome patients. Eur. Rev. Med. Pharmacol. Sci..

[24] Rotterdam ESHRE/ASRM-Sponsored PCOS Consensus Workshop Group (2004). Revised 2003 consensus on diagnostic criteria and long-term health risks related to polycystic ovary syndrome (PCOS). Hum. Reprod..

[25] Ferraretti AP (2011). ESHRE consensus on the definition of ‘poor response’ to ovarian stimulation for in vitro fertilization: the Bologna criteria. Hum. Reprod..

[26] Johnson NP (2017). World Endometriosis Society consensus on the classification of endometriosis. Hum. Reprod..

[27] Besmer P (1991). The kit ligand encoded at the murine Steel locus: A pleiotropic growth and differentiation factor. Curr. Opin. Cell Biol..

[28] Manova K (1993). The expression pattern of the c-kit ligand in Gonads of mice supports a role for the c-kit receptor in oocyte growth and in proliferation of spermatogonia. Dev. Biol..

[29] Motro B, Bernstein A (1993). Dynamic changes in ovarian c-kit and Steel expression during the estrous reproductive cycle. Dev. Dyn..

[30] Franco-Murillo Y (2015). Unremitting cell proliferation in the secretory phase of eutopic endometriosis: involvement of pAkt and pGSK3β. Reprod. Sci..

[31] Dewailly D (2016). Interactions between androgens, FSH, anti-Müllerian hormone and estradiol during folliculogenesis in the human normal and polycystic ovary. Hum. Reprod. Update.

[32] Fu Y-X (2020). Anti-Müllerian hormone regulates stem cell factor via cAMP/PKA signaling pathway in human granulosa cells by inhibiting the phosphorylation of CREB. Reprod. Sci..

[33] Osuga Y (2000). Stem Cell Factor (SCF) concentrations in peritoneal fluid of women with or without endometriosis. Am. J. Reprod. Immunol..

[34] Osuga Y (2010). Current concepts of the pathogenesis of endometriosis. Reprod. Med. Biol..

[35] Osuga Y, Hirota Y, Taketani Y (2008). Basic and translational research on proteinase-activated receptors: Proteinase-activated receptors in female reproductive tissues and endometriosis. J. Pharmacol. Sci..

[36] Hwang J-H (2013). Identification of biomarkers for endometriosis in eutopic endometrial cells from patients with endometriosis using a proteomics approach. Mol. Med. Rep..

[37] Lukaszuk K (2014). Anti-Müllerian hormone (AMH) is a strong predictor of live birth in women undergoing assisted reproductive technology. Reprod. Biol..

[38] Liss J (2018). Effect of next-generation sequencing in preimplantation genetic testing on live birth ratio. Reprod. Fertil. Dev..

[39] Balaban B (2011). The Istanbul consensus workshop on embryo assessment: Proceedings of an expert meeting. Hum. Reprod..

